# Subarctic sugar kelp (*Saccharina latissima*, Phaeophyceae) summer productivity and contribution to carbon budgets

**DOI:** 10.1111/jpy.13525

**Published:** 2024-11-25

**Authors:** Stéphanie Roy, Christian Nozais, Ladd E. Johnson, Fanny Noisette

**Affiliations:** ^1^ Institut des Sciences de la Mer Université du Québec à Rimouski, and Québec‐Océan Rimouski Québec Canada; ^2^ Département de biologie, chimie et géographie Université du Québec à Rimouski, and Québec‐Océan Rimouski Québec Canada; ^3^ Département de biologie Université Laval, and Québec‐Océan Québec Québec Canada

**Keywords:** biomass production, brown macroalgae, growth, in situ photorespirometry, primary productivity

## Abstract

Kelp forests are known to be very productive ecosystems and constitute a central component of the marine carbon cycle in coastal areas. Nevertheless, crucial carbon‐related data are missing to be able to include them properly in carbon budgets. A thorough understanding of the kelp contribution to the carbon cycle is especially important in regions prone to experiencing strong seasonal fluctuations in environmental conditions, such as subarctic regions. This study aimed to quantify primary productivity through growth rates and oxygen fluxes of a dominant kelp species in subarctic regions, *Saccharina latissima*, and to link oxygen fluxes to environmental parameters. Our results showed that strong primary productivity oxygen fluxes coincided with high light levels in July and most of August, while growth rates stayed similar all summer. An overall decline in all primary productivity proxies happened from late August, suggesting a seasonal slowing down of *S. latissima* metabolism. The estimated quantity of carbon stored in tissue during growth represented from 6% to 28% of the gross primary productivity. Further research is needed to explore how and how much carbon transits through living kelp tissue in different seasons, to better understand the contribution of subarctic kelp to coastal carbon budgets.

**Abbreviations**
BL: blade length
BP: biomass production
DA: dominance analysis
G: growth
GPP: gross primary productivity
NPP: net primary productivity
R: respiration

## INTRODUCTION

Kelps are large brown ecosystem engineer macroalgae forming widely distributed underwater forests from temperate to polar regions (Jones et al., 1994; Steneck et al., 2002). Kelp forests cover more than 20% of the world's coastlines, corresponding approximately to 2.03 × 10^6^ km^2^ (Filbee‐Dexter et al., 2016; Jayathilake & Costello, 2020; Pessarrodona et al., 2023). They serve as essential habitats for a wide range of marine species, have a critical role in supporting high biodiversity (Teagle et al., 2017), and sustain economically important species, including commercially valuable fish and invertebrates (Smale et al., 2013; Wernberg et al., 2011). Renowned for their high productivity in coastal oceans (Duarte et al., 2022; Smith, 1981), kelp forests have high capacities of carbon fixation via photosynthesis and carbon storage in living tissues (Filbee‐Dexter & Wernberg, 2020; Krause‐Jensen & Duarte, 2016; Mann, 1973). Kelp forests have been proposed to be added to the blue carbon ecosystem trio (seagrass beds, saltmarshes, and mangroves) as a serious nature‐based solution for contributing to climate change mitigation (Duarte et al., 2022; Hill et al., 2015; Krause‐Jensen et al., 2018; Lovelock & Duarte, 2019). However, the efficiency of kelp forests as blue carbon ecosystems or nature‐based solution to mitigate climate change is under an ongoing debate (Boyd et al., 2022; Filbee‐Dexter et al., 2023; Gallagher, 2023; Gallagher et al., 2022; Hurd et al., 2024; Ross et al., 2023; Troell et al., 2024), and as such, kelp forests are often omitted in global carbon budgets (Duarte, 2017; Hurd et al., 2022). This debate stems primarily from the lack of carbon‐related data in many regions, for various species, at different seasons (Pessarrodona et al., 2023), as well as from a limited understanding of kelp‐related carbon fluxes in local and global systems. The key components that lack comprehensive data include atmosphere–ocean carbon exchange, detritus dynamics (e.g., export, deposition, and decomposition rates), and the magnitude and distribution of kelp primary productivity (Filbee‐Dexter et al., 2023; Gilson et al., 2023; Hurd et al., 2024; Pessarrodona, Assis, et al., 2022; Ross et al., 2023; Smale et al., 2020). To gain a comprehensive understanding of the kelp contribution to the carbon cycle, it is crucial to thoroughly understand kelp carbon uptake through photosynthesis (i.e., fixation) and its conversion into organic matter as kelp biomass (i.e., assimilation; Pessarrodona et al., 2023).

Past studies on kelp primary productivity in North Atlantic have been mostly focused on temperate regions (e.g., Gilson et al., 2023; Hatcher et al., 1977; Krumhansl & Scheibling, 2011). However, arctic and subarctic kelp experience seasonal variations that diverge greatly from cold‐temperate regions: Low nutrient availability lasts only during the short summer period, light intensity and quality are overall lower at higher latitudes because of ice cover and clouds (Tremblay et al., 2015), and temperature varies from freezing point to thermal threshold for kelps (Muth et al., 2019), leading to distinctive growth patterns and productivity rates that have been poorly studied in subarctic regions compared to temperate ones (Pessarrodona, Filbee‐Dexter, et al., 2022). Indeed, the growth period is shorter at high latitudes and growth rates are usually slower at low temperatures for temperate species such as *Saccharina latissima* (Rinde & Sjøtun, 2005; Smale et al., 2020). Furthermore, arctic and subarctic regions may constitute a future refuge for kelp species experiencing a poleward shift due to ocean warming (Khan et al., 2018, Wilson et al., 2019), and it becomes all the more relevant to study these kelp populations and their primary productivity.

Traditionally, primary productivity has been assessed by standing stock estimation (Brady‐Campbell et al., 1984; Gilson et al., 2023; Hatcher et al., 1977; Mann, 1972; Smale et al., 2016), growth measurement, or change in biomass (de Bettignies et al., 2013; Krumhansl & Scheibling, 2011; Pessarrodona, Foggo, & Smale, 2018). These measurements provide an integrated picture of the carbon stored by kelp; however, they may not fully capture the true capacity of kelps to fix carbon through primary productivity, as they do not consider carbon fluxes (e.g., carbon fixation by photosynthesis and release of dissolved organic carbon). Direct measurements that can closely capture the fine temporal variability in primary productivity related to environmental changes are crucial in order to have a clear picture of the processes underlying carbon fixation, assimilation, and storage. In recent years, photorespirometry, a technique using oxygen fluxes and conversion with photosynthetic quotients, has been used to measure carbon fixation (Gevaert et al., 2011; Rodgers et al., 2015; Rodgers & Shears, 2016; Tait et al., 2014; White et al., 2021; White & Davoult, 2022). This technique yields an accurate quantification of carbon fluxes that can be used to assess temporal variability associated with local environmental conditions. In situ underwater photorespirometry reduces artifacts associated with laboratory studies, such as underestimation of primary productivity (Rodgers & Shears, 2016, White et al., 2021, White & Davoult, 2022). It also permits underpinning physiological responses to environmental conditions as they naturally occur, for example, under changing light conditions (e.g., sun flecks).

Only a few studies have used photorespirometry techniques on whole individuals with chambers replicating adequately natural conditions (e.g., natural position of the seaweed and water flow in the chamber similar to in situ hydrodynamics; Rodgers et al., 2015, Rodgers & Shears, 2016, Blain & Shears, 2019, White et al., 2021, White & Davoult, 2022). These studies have focused mostly on widely distributed temperate species such as *Laminaria hyperborea* and *Ecklonia radiata*. To our knowledge, only one study has examined kelp primary productivity using photorespirometry methods in the Arctic (Borum et al., 2002) and none in subarctic regions (Pessarrodona, Filbee‐Dexter, et al., 2022). Furthermore, combining in situ underwater photorespirometry with integrated measurements such as growth and biomass production can give a more accurate portrait of productivity (e.g., carbon assimilation) and carbon dynamics (Hatcher et al., 1977; Pessarrodona, Filbee‐Dexter, et al., 2022).

The sugar kelp *Saccharina latissima* (formerly *Laminaria saccharina* and *S. longicruris*: McDevit & Saunders, 2010) is a species widely distributed from temperate to arctic regions (Goldsmit et al., 2021; Jayathilake & Costello, 2020); however, in recent years, its range has been observed to decrease in the south (Feehan et al., 2019; Filbee‐Dexter et al., 2016; Moy & Christie, 2012) and increase in the north (Bartsch et al., 2016; Krause‐Jensen et al., 2012, 2020). *Saccharina latissima* distributional data are still very limited in Eastern Canada (Merzouk & Johnson, 2011), even though it is one of the most important kelp forest‐forming species on the Atlantic western coast (Teagle et al., 2017) and is often the dominant species, such as in the St. Lawrence estuary and Gulf of St. Lawrence (Himmelman et al., 1983; Roy et al., 2023; Tamigneaux & Johnson, 2016). It has also been identified as an excellent model to forecast future distribution and survival in the face of climate change (Diehl et al., 2024; Goldsmit et al., 2021). Given its important ecological roles, direct measurements of its productivity would help to better portray the role of *S. latissima* in the carbon cycle of subarctic coastal zones. This study assessed its productivity by measuring a full suite of metrics, including in situ measures of photosynthesis and respiration, individual growth rates, and cumulative changes in individual size and biomass during the summer. We predicted that (1) net primary productivity and growth rates would be highest during June and July when light and temperature were highest; (2) blades would be longest and biomass production highest in early August after high growth rates during June and July; and (3) all metrics would decrease from September, probably driven by a seasonal decrease in irradiance and daylight.

## MATERIALS AND METHODS

### Study site and environmental parameters

**FIGURE 1:**
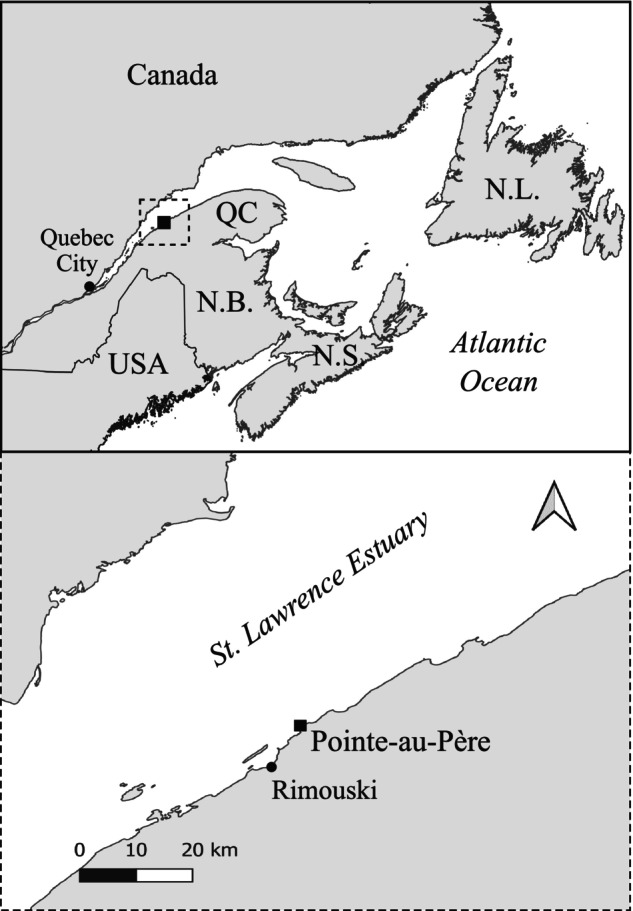
Location of the study area. NB, New Brunswick; NL, Newfoundland and Labrador; NS, Nova Scotia; QC, Québec; USA, United States.

The study site was located on the south shore of the lower St. Lawrence maritime estuary in Pointe‐au‐Père, Rimouski, Quebec, Canada (48°31′ N, 68°27′ W; Figure 1), in the transition zone between boreal and subarctic regions (Merzouk & Johnson, 2011). Although both boreal and subarctic seaweed species are present, the species composition is more subarctic than boreal, particularly because of the absence of the red alga *Chondrus crispus*, which usually represents the thermographic boundary of boreal regions (Adey & Hayek, 2011). Furthermore, the environmental conditions (e.g., light, temperature, and arctic currents) resemble subarctic conditions, as there is ice cover for more than 3 months a year, ice scour during winter on the shallow seaweed assemblages, and a relatively cold year‐round water temperature, with an average temperature of 4°C (Adey & Hayek, 2011; Galbraith and Fisheries and Oceans Canada, 2023).

*Saccharina latissima* dominated the kelp forest at the site, which was a subtidal rock bed located on a gently sloping bottom at depths ranging from 1 to 5 m depending on the tides (mean low water of 2.2 m; maximal tidal range of 4.7 m). The kelp forest extended parallel to the shore in a narrow band reaching up to 11 m wide. During the duration of the study (May to September), data loggers for conductivity (U24‐002‐C, Hobo® conductivity logger, Onset, Computer corporation, MA, USA) and temperature/light (UA‐002‐64, Onset, Hobo® Pendant temp/light) were deployed on a mooring close to the kelp forest at the same depth. The logging interval was 60 min, and sensors were cleaned every 2 weeks by divers to remove any biofouling.

### Growth assessment

#### Growth rate

The growth of *Saccharina latissima* was measured from May to August 2022. On May 20, 40 individuals were tagged by divers with cable ties and identified with numbered plastic tags attached loosely around the stipe. On May 25, these individuals were measured (length and width) and punched with distinctive heart‐shaped holes at 10 cm and 15 cm above the meristematic transition zone between the blade and the stipe (Parke, 1948). Blade length and the distance between these holes and stipe–blade junction were measured periodically thereafter, respectively five and four times over summer. The interval between each sampling varied from 15 to 36 days. New holes were punched at each visit as the older ones were often lost or deformed due to apical erosion and grazing. At each visit, new individuals were tagged to replace individuals lost by dislodgment, in order to maintain approximately 40 tagged individuals at all times. Individual linear growth rates were adjusted to the number of days between sampling events.

#### Calculation of biomass production

The method from Krumhansl and Scheibling (2011) was adapted to calculate kelp biomass production during summer. Five to 10 entire individuals were collected by cutting the stipe a few centimeters above the holdfast on three occasions: May 27, August 5, and September 12. Each time, an ~5‐cm‐long segment was sampled in the middle of blade of each individual. The weight and length of each segment were measured before being placed in burnt aluminum foil at −80°C for further carbon and nitrogen analysis. For each sampling event, the *B*_base_ was calculated as the average of individual segment ratio: weight wet (WW) in g/length in cm (May: *n* = 7, August: *n* = 5, and September: *n* = 10). The following equation was then used to estimate daily biomass production (BP; g WW · d^−1^) by multiplying the daily growth (G; cm) with the *B*_base_. The biomass production in g WW · d^−1^ could then be transformed into g dry weight (DW) · d^−1^ by using the mean WW:DW ratio calculated from the blades collected for establishing allometric relationship (*n* = 20 to 52 depending on the sampling event, Appendix S1 and Figure S1 in the Supporting Information). The ratios were respectively, 10.5, 7.3, and 9.0 for May, August, and September, respectively.

$$\mathrm{BP}=G\times B_{\text{base}}\times \text{ratio}\;\mathrm{WW}:\mathrm{DW}$$

The *B*_base_ varied among months, and each was associated with a specific growth period to estimate biomass production. As to such, the *B*_base_ coefficient for May 27 was associated with the June growth period, the *B*_base_ coefficient from the August 5 sampling was associated with July and early August growth and the *B*_base_ coefficient for September 12 was used for late August growth.

### In situ primary productivity measurements

**FIGURE 2:**
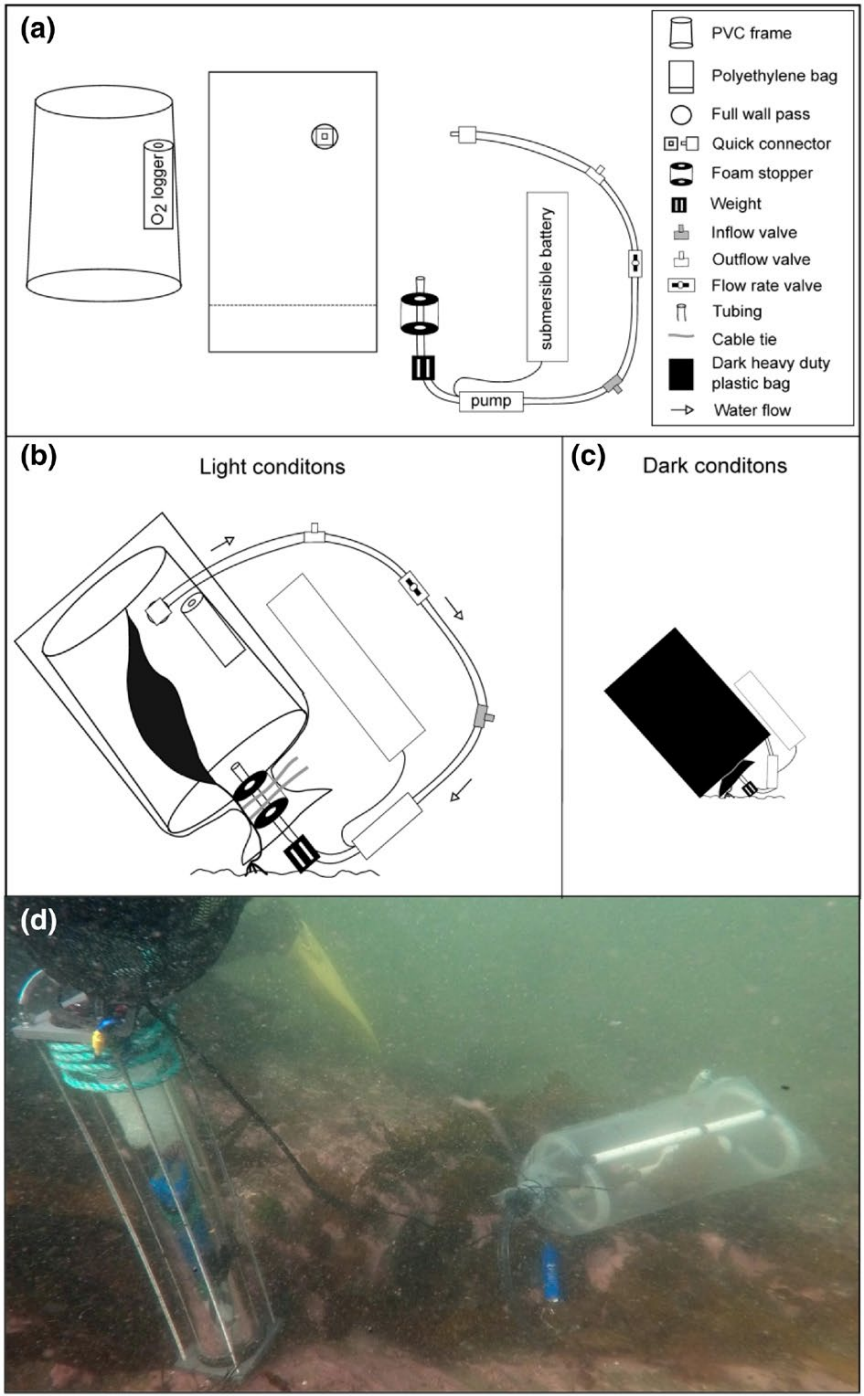
Incubation chamber components (a) and the in situ incubation procedure (b, c) and underwater deployment of the incubation chamber (d). The chamber was hermetically closed around the foam stopper and the stipe, after what it was filled in with the surrounding water by the inline pump within the tubing and powered by the battery. Once filled, the inflow and outflow valves were closed and the water circulated in a clockwise manner. The water exited the main chamber into the tubing from the top corner where the oxygen logger was installed, which took measurements every minute. For dark conditions, an opaque plastic bag was attached over the whole chamber. [Color figure can be viewed at wileyonlinelibrary.com]

An in situ photorespirometry technique was used to measure respiration and photosynthesis rates on individual kelp blades during four different periods: from June 12 to 25 (*n* = 8), from July 26 to August 3 (*n* = 6), from August 19 to 25 (*n* = 6), and from September 20 to 26 (*n* = 6). Individuals ranging from 40 to 140 cm in blade length were selected for incubations, as this size range was large enough to produce an oxygen signal in less than an hour but small enough to minimize any self‐shading in the incubation chamber (Figure 2).

#### Description of the incubation chamber

The incubation chamber used to measure primary productivity in situ was composed of a clear polyethylene bag (81 × 33 cm, 200 μm thick) with a recirculation system. It included an internal cylindrical frame (20–22 cm width and 50 cm height) made of a combination of rigid and flexible plastic pipes with the lower end of the frame being slightly wider than the upper one for easier insertion into the bag (Figure 2). The frame shaped the chamber system to provide the blade enough space to assume a natural orientation and avoid self‐shading. It also served as a support for an oxygen and temperature logger (miniDOT logger, Precision Measurement Engineering, Vista, CA, USA), which was attached to the upper part of the frame. The bag was connected to tubes attached to an external inline pump (iL200, Rule), which created a loop that circulated water from the bottom to the top of the chamber at a rate of 5 L · min^−1^. Inflow and outflow valves located on the tubing allowed for controlling the water flow and also for the initial filling of the chamber with the surrounding water. To hermetically enclose the blade in the chamber, a soft foam stopper was wrapped around the stipe just below the junction with the blade. Given that the majority of primary productivity occurs in the kelp blade (Gevaert et al., 2011), only this part of the kelp was inserted in the chamber, and kelp remained attached to the substratum. Volume of kelp blades was estimated to a maximum of 2% of the total volume of the chamber. The plastic bag was then secured around the foam stopper with two cable ties at a pre‐marked line to keep the volume close to 20 L (depending on the volume of the algae) and to prevent any water exchange with the surroundings. (Chambers were indeed hermetically sealed as tested in trials with oxygen‐enriched water beforehand.) A 1‐kg weight was attached just below the foam stopper to keep the chamber on the substratum at a 45° angle, similar to the natural position of kelp (Steneck et al., 2002; Wernberg & Filbee‐Dexter, 2019). To create dark conditions, an opaque black plastic bag was used to cover the entire chamber and was secured with cable ties to the tubing.

#### Incubation procedure

Once sealed, the chamber was filled with surrounding water before closing the valves to hermetically seal the chamber. The incubations started under dark conditions to decrease the oxygen concentration in the chamber and avoid oxygen super‐saturation. After 40 min, the dark plastic bag was removed and a 50‐min light incubation started, which included an operationally defined 10‐min period of light acclimation followed by a 40‐min period when oxygen production rates were constant (Noël et al., 2010). The chamber was then removed and the blade was detached by cutting the stipe a few centimeters above the holdfast and kept for later morphological measurements of length, width, and dry weight. During both incubation periods, the miniDOT logger in the chamber measured dissolved oxygen concentration (mg O_2_ · L^−1^) and temperature (°C) every minute for the whole incubation process. Two types of temperature/light sensors (miniPAR logger, Precision Measurement Engineering, Vista, CA, USA, and UA‐002‐64, Onset, Hobo® Pendant temp/light, Onset, Computer Corporation, MA, USA) were also deployed in proximity to the incubation chambers (approximately 1–10 m) and at similar depths to measure the irradiance (μmol photons · m^−2^ · s^−1^ and lux, respectively) every minute during the incubation. It is to be noted that we only acquired the miniPAR logger from August; therefore we had only the HOBO as light loggers for the month of June and July.

#### Net primary production and respiration calculation

Rates of net primary production (NPP) and respiration (R) were calculated from the linear slope of dissolved oxygen concentration as a function of time, during light and dark incubations, respectively. Dissolved oxygen concentration was linear throughout incubations, indicating that no nutrient limitation or oxygen super‐saturation occurred in the chamber (Noël et al., 2010). Slopes were calculated from the entire 40‐min incubation period except on one occasion when a battery stopped, and only an 11‐min period was linear. Net primary production and R rates were standardized for kelp dry biomass (as a linear relationship between NPP and DW proved significant) and corrected by the chamber volume of 20 L (μmol O_2_ · g^−1^ DW · h^−1^). The gross primary productivity (GPP) was calculated by summing the NPP and the absolute value of R, with the caveat that respiration measured under dark conditions is not truly equal to respiration during light conditions (Zou et al., 2011).

### Carbon calculations

#### Carbon rates from incubations

Carbon rates can be calculated from oxygen rates by converting O_2_ into CO_2_ with the photosynthetic quotient (PQ), which represents the relationship between carbon fixation and oxygen production during photosynthesis. Typically, a 1:1 ratio can be used (Hansen et al., 2011), although species‐specific ratios can be calculated (Gerard, 1988; Iñiguez et al., 2016). Here, PQ = 1.13 was used for *Saccharina latissima* in a shallow and turbid environment (Gerard, 1988).

$$\text{Carbon rate}=\frac{\left(\text{Oxygen rate}\times 1.13\right)}{1000}\times 12.01\times \text{photoperiod}$$

Carbon rates in μmol C · h^−1^ were converted into mg C · h^−1^ by using the carbon molar mass of 12.01 g · mol^−1^. Rates were then converted into mg C · d^−1^ by multiplying by the photoperiod and dividing by 1000. Photoperiods were obtained from the light/temperature in situ loggers and were of 15.5 h for June and July, 14.5 h for August, and 12.5 h for September.

#### Blade carbon content

Carbon assimilation calculations were realized using the blade carbon content. Previously dried and frozen ~5‐cm‐long pieces of kelp (see Calculation of biomass production) were freeze‐dried (48 h, Labcono, USA), ground, enclosed into the tin capsules, and analyzed with an elementary analyzer (ECS 4010, Costech Analytical Technologies, Inc., USA) to obtain carbon and nitrogen contents. Measured carbon contents (May: *n* = 7, August: *n* = 5, and September: *n* = 10) were then averaged for each period (C_avg_) and used to convert biomass production into carbon assimilation C_assim_.

$$C_{\text{assim}}=\mathrm{BP}\times C_{\mathrm{avg}}$$

where C_assim_ is the rate of carbon assimilation in mg C · d^−1^, BP is the biomass production in mg DW · d^−1^, and C_avg_ is the mean carbon content in mg C · mg^−1^ DW.

The mean C:N ratio from the blade tissues was calculated for May, August, and September by dividing the carbon (mg) and nitrogen (mg) with their respective molar masses, before diving those values with one another. The average ratio was therefore expressed in mol:mol.

### Data analysis

All analyses were conducted and graphics were created with the R Statistical Software v4.3.0 (R Core Team, 2023; Wickham, 2016; Wickham et al., 2023). As growth and blade length were followed on the same individuals over the summer, analyses of variants (ANOVAs) with repeated measurements were performed to test for differences in growth, biomass production rates, and blade length between months, with the individual as a random factor to account for temporal pseudoreplication. The emmeans package was used to perform the post hoc tests with the Bonferroni correction to determine which specific months were significantly different from each other (Kruchek & Lenth, 2022). One‐way ANOVAs followed by emmeans with the Bonferroni correction were used to test the difference in primary productivity rates (GPP and R) between months. All ANOVA assumptions were tested with Shapiro–Wilk and Levene tests. Data that did not fulfill the normality assumptions were transformed (square‐root transformation for blade length).

Multiple regression models were built to assess the effect of environmental variables (light, salinity, and temperature) on oxygen fluxes. Temperature and salinity values used for building relationships were the averages from the day of sampling. Salinity was calculated from the conductivity data using a conversion table (Aminot & Kérouel, 2004). As light levels can vary quickly over a short time span (i.e., due to clouds), light levels considered were calculated as the average irradiance for the time of the incubation, recorded with the miniPAR and HOBO light sensor (see Section Incubation procedure). To convert the units from lux to μmol photons · m^−2^ · s^−1^, a calibration coefficient of 0.038 was calculated from simultaneous recordings of the light sensors deployed during primary production incubation (see Section Incubation procedure above). The coefficient was calculated from a regression built from in situ measurements under multiple light conditions (high and low turbidity, cloudy and sunny sky), allowing the application of this calibration over a range of environmental conditions (Long et al., 2012). Multicollinearity between predictors, used for multiple regression models, was examined using a correlation plot and variance inflation factor (VIF; package corrplot; Wei & Simko, 2021). A predictor was removed if the VIF was higher than five, indicating a high correlation (Jou et al., 2014). The best models were selected using the Akaike information criterion, and the linear regression assumptions were tested with diagnostic plots, as well as Shapiro–Wilk and Breusch–Pagan tests on the model when uncertain of the diagnostic plots (package lmtest; Zeilis & Hothorn, 2002). Dominance analysis (DA) was then performed to determine the percentage of variance explained by each significant predictor (package domir; Luchman, 2023).

## RESULTS

### Growth rates

**FIGURE 3:**
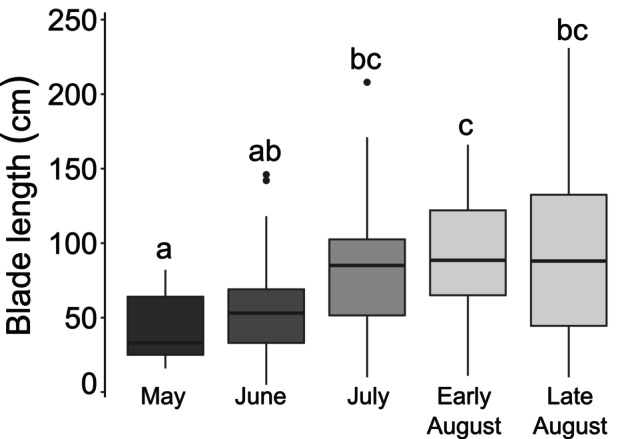
Blade lengths from May to August. Shades of gray represent the months and the letters represent significant differences between months, *n* = 13, 37, 39, 38, 19, for each period.

The blade length was significantly different among months with the shortest blades recorded in May (for all following, mean ± SE; 41.2 ± 6.3 cm) and the longest blades in August (92.5 ± 6.6 cm and 88.3 ± 13.7 cm for early and late August, respectively; Figure 3, Table 1).

**TABLE 1:** Detailed statistical analysis.

Test	Response variable	Explanatory variables	*p*‐value	*R* ^2^
ANOVA	BL	Month + (1|ind)	Month <0.05	–
Emmeans			June–July June–early August June–late August Early August–late August (1|ind) < 0.05	
ANOVA	G	“ ”	Month: 0.15 1|ind: 0.71	–
ANOVA	BP	“ ”	Month<0.05 June–July June–early August July–late August Early August–late August 1|ind <0.05	
Emmeans				
ANOVA	GPP	Month	<0.05	–
emmeans			June–July June–August September–July September–August	
ANOVA	R	“ ”	Month: 0.084	–
Multiple regression	GPP	Temperature + salinity + light	Light <0.05	0.63
Multiple regression	R	“ ”	Temperature <0.05 Salinity <0.05	0.37

Abbreviations: BL, blade length; BP, biomass production; G, growth; GPP, gross primary productivity; NPP, net primary productivity; R, respiration.

**FIGURE 4:**
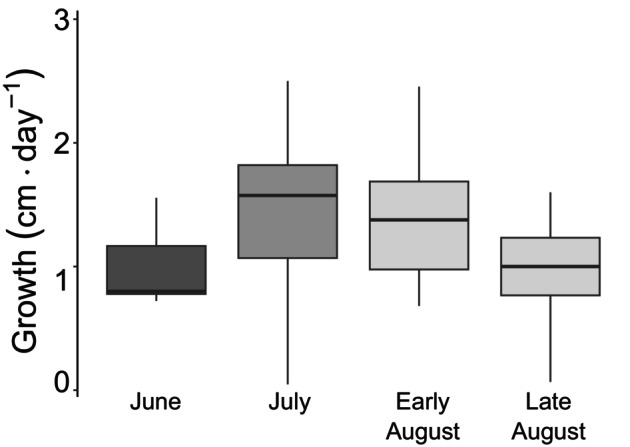
Growth (cm · d^−1^) from June to August. Shades of gray represent the months, *n* = 5, 22, 18, 7, for each period, respectively.

Linear growth rates did not differ significantly among months (Table 1). Growth rates were the highest in early August (1.41 ± 0.13 cm · d^−1^) and July (1.38 ± 0.13 cm · d^−1^) and the lowest in late August (0.95 ± 0.19 cm · d^−1^; Figure 4; Table 1).

**FIGURE 5:**
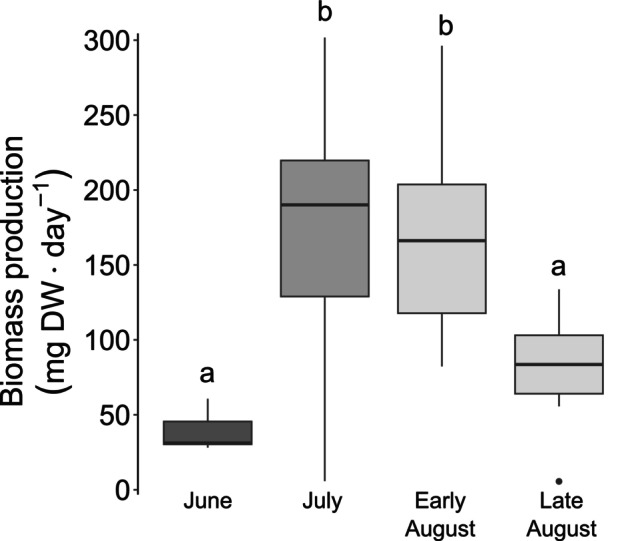
Biomass production (mg DW · d^−1^) from June to August. Shades of gray represent the months and different letters represent significant differences. *n* = 5, 22, 18, 7, for each period, respectively.

The mean biomass production was significantly different among months with the highest biomass production in early August, closely followed by July (170 ± 15 mg · d^−1^, 167 ± 15 mg · d^−1^, respectively; Table 1; Figure 5). The lowest biomass production occurred in June (39 ± 6 mg · d^−1^).

No effect of individuals was significant on linear growth rate but was significant for biomass production and blade length (Table 1).

The C:N ratio was at its lowest in May (mean ± *SD*, 14.1 ± 0.75 mol:mol) and increased during the summer (August; 20.9 ± 0.10 mol:mol), being at its highest in September (22.7 ± 0.6 mol:mol).

### Primary productivity

**FIGURE 6:**
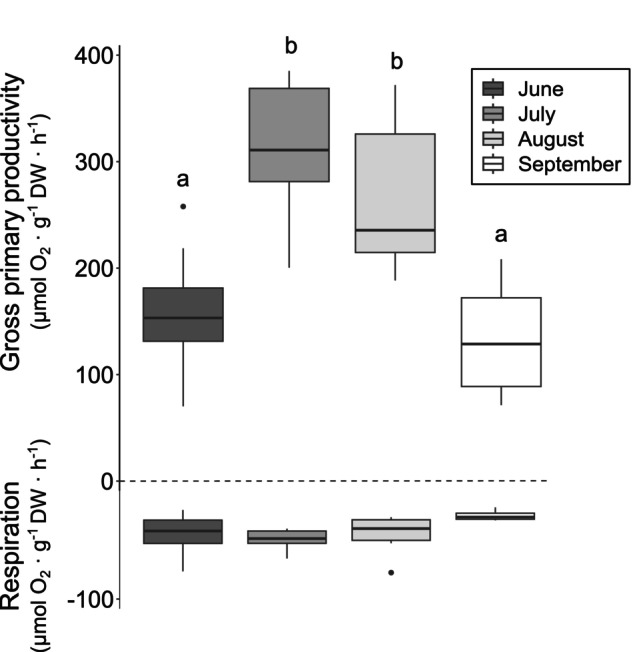
Gross primary production (μmol O_2_ · g^−1^ DW · h^−1^) and respiration (μmol O_2_ · g^−1^ DW · h^−1^) during summer months. *n* = 8, 6, 6, 6. Different letters show significant differences between months.

The gross primary productivity ranged from 70 to 385 μmol O_2_ · g^−1^ DW · h^−1^, and the respiration from −15 to −75 μmol O_2_ · g^−1^ DW · h^−1^ (Figure 6). The GPP was significantly higher in July and August, compared with June and September (Table 1; Figure 6), whereas the respiration was similar among summer months.

### Links between primary productivity and environmental variables

The water temperature was warmest in early August, the irradiance strongest in July, and the salinity highest in September (Table 2). For gross primary productivity (GPP), significant predictors only included the average light during the in incubation, and the model accounted for 44% of the variance. Regarding respiration, the optimal model, which included average daily temperature and salinity as significant predictors, explained 37% of the variance. Specifically, temperature (DA; 21%) emerged as the strongest predictor, closely followed by salinity (DA; 16%).

**TABLE 2:** Temperature, irradiance, and salinity (mean ± SD) during the summer months at Pointe‐au‐Père, Québec, Canada.

	Temperature (°C)	Irradiance (μmol photons · m^−2^ · s^−1^)	Salinity
June	8.0 ± 2.0	227 ± 356	20.2 ± 1.5
July	9.7 ± 1.5	344 ± 517	21.4 ± 1.4
Early August	10.1 ± 1.2	333 ± 402	22.0 ± 1.2
Late August	9.1 ± 1.6	296 ± 365	23.3 ± 1.4
September	8.5 ± 0.9	255 ± 333	23.8 ± 0.9

*Note*: The irradiance data might be overestimated as we observed some anomalies in the daily variation of irradiance, always corresponding with low tides and the solar zenith. We associated those peaks in the data with the type of data logger we used. Specifically, the Hobo logger was planar and thus measured the light exactly above it, explaining the anomalous peaks we observed in the data.

### Scaling up carbon estimations

The mean daily productivity of the *Saccharina latissima* forest at Pointe‐au‐Père was the highest in July and the lowest in June for all metrics except for biomass production, which was the highest in early August (Table 3). The mean biomass production (mg C d^−1^) represented at most 28% of the GPP (early August) and the mean carbon loss by respiration represented at most 22% of the GPP (June), assuming that the GPP rate measured was constant over the day (Figure 1).

**TABLE 3:** A Individual carbon rates (mg C · d^−1^) for primary productivity and assimilation of carbon by biomass production via growth of *Saccharina latissima* during summer months at Pointe‐au‐Père, Canada.

	GPP (mg C · d^−1^)	NPP (mg C · d^−1^)	R (mg C · d^−1^)	BP (mg C · d^−1^)
June	160.6 ± 44.4	125.3 ± 43.3	−35.2 ± 5.6	10.9 ± 1.7
July	353.2 ± 42.8	300.2 ± 35.5	−53.0 ± 8.5	49.1 ± 4.5
Early August	181.2 ± 34.8	156.2 ± 32.5	−25.0 ± 3.5	50.2 ± 4.4
End August				20.0 ± 3.9
September	181.9 ± 18.0	148.0 ± 19.6	−33.9 ± 3.5	NA

Abréviations: BP, biomass production; GPP, gross primary productivity; NPP, net primary productivity; R, respiration.

## DISCUSSION

This study aimed to estimate the productivity of the kelp *Saccharina latissima* in a subarctic environment and assess the effect of environmental parameters on its primary productivity by measuring a full suite of metrics including oxygen fluxes, growth rates, and cumulative changes in individual size and biomass. Our results showed relatively constant growth rates across the summer season. Conversely, GPP peaked in July and August and was correlated with the highest irradiance measured in situ. The estimated biomass production (derived from growth measurements) closely mirrored variation in primary productivity with peak rates precisely coinciding with periods characterized by elevated primary productivity rates. A decrease in productivity was noticeable in late August and September, hinting at a slowing down in metabolism toward the end of summer.

### High productivity rates of *Saccharina latissima* in subarctic environments

Net primary productivity of *Saccharina latissima* in summer at Pointe‐au‐Père (ranging from 8 and 343 μmol O_2_ · g^−1^ DW · h^−1^) and the respiration (ranging from −16 to −75 μmol O_2_ · g^−1^ DW · h^−1^) surpassed the summer productivity of the same species measured in Nova Scotia (Hatcher et al., 1977) and Greenland (Borum et al., 2002), where the NPP and respiration measured fell into the low end of the range observed in our study (Table 4). These differences could be driven by the acclimation of *S. latissima* to local conditions, with adjustments observed to be specific to each site, making it difficult to generalize these adaptations from one population to the entire species complex (Diehl et al., 2024; Diehl & Bischof, 2021; Spurkland & Iken, 2012). These contrasts in metabolism can result from differences in experimental conditions (e.g., light availabilities) for measuring primary production or in the part of the alga used for measurements. Relative to other species, the net primary productivity of *S. latissima* in our study was three to four times higher compared with other kelp species examined in other regions, such as *Laminaria hyperborea* in Brittany and Norway or *Ecklonia radiata* in New Zealand, even when measured with similar photorespirometry devices (in gray; Table 4). Even if measurements were made under a similar range of irradiance (e.g., 0 to 515 μmol photons · m^−2^ · s^−1^, this study; 0 to 510 μmol photons · m^−2^ · s^−1^, Rodgers et al., 2015; and 0 to 650 μmol photons · m^−2^ · s^−1^, White et al., 2021), kelp metabolism can be very species‐specific (Abdullah & Fredriksen, 2004; Bartsch et al., 2008; Iñiguez et al., 2016; Staehr & Wernberg, 2009) and can also be influenced by local temperature regimes (Andersen et al., 2013), hydrodynamics (Gilson et al., 2023), or desalinization episodes (Monteiro et al., 2021). Our measurements of respiration for *S. latissima* were up to 15 times higher than other species from the Laminariaceae family (Table 4). These significantly higher respiration rates could either be a unique characteristic of *S. latissima* in the St. Lawrence estuary or an indication of stress (Harley et al., 2012; Tait, 2014). This stress might be due to temperatures approximately 2°C higher than the monthly averages, the temperature which explained 21% of the variation in respiration rates in the dominance analysis above.

**TABLE 4:** In situ kelp productivity in μmol O_2_ · g^−1^ DW · h^−1^.

Region	Species	Net primary productivity	Respiration	Season	Depth (m)	References
Québec	*Saccharina latissima*	48 to 343 or *0.37 to 2.17* or **5 to 56**	−16 to −75 or *−0.15 to −0.58* or **−2 to −11**	Summer	1–5	This study
Nova Scotia	*S. latissima*	*−0.04 to 0.54*	*−0.07 to −0.40*	All year	10	Hatcher et al. (1977)
Greenland	*S. latissima* (disc only)	21 to 85*	−3 to −21	Summer	2–20	Borum et al. (2002)
Brittany	*Laminaria hyperborea*	−20 to 80	−8.54 ± 1.98	Summer Autumn	4 ± 1	White et al. (2021)
Norway	*L. hyperborea*	**3 to 20**	**−2 to −5**	Spring Summer Autumn	<30	Abdullah and Fredriksen (2004)
New Zealand	*Ecklonia radiatia*	40 to 110*	−7 to −16	All year	8–10	Blain and Shears (2019)
New Zealand	*E. radiata*	49 to 114	−13.5 ± 2.8	Winter	6 or 14	Rodgers et al. (2015)

*Note*: Productivity measurement in bold in μmol O_2_ · g^−1^ WW · h^−1^ and italics in μmol O_2_ · cm^−2^ · h^−1^. The conversion to μmol O_2_ · cm^−2^ · h^−1^ is explained in Appendix S1. Rows in gray used very similar incubation chamber. The asterisk represents the maximal net primary productivity as maximum photosynthetic rates (P_max_)

That high net primary productivity and respiration rates measured in kelp in our study in the midst of summer temporally bordered with a seasonal low of those measurements, underlines the short time frame to benefit from favorable environmental conditions (e.g. light, temperature) that has been observed in higher latitudes in Alaska and Norway (Rinde & Sjøtun, 2005; Spurkland & Iken, 2011, 2012). The yearly peak in primary productivity was likely captured during our study, at least in terms of oxygen fluxes, given that environmental conditions during other seasons are not ideal for photosynthesis given the subarctic climate. Therefore, *Saccharina latissima* at our site seemed to maximize primary production during the short window when environmental conditions are optimal during summer. Year‐round oxygen flux measurements on *S. latissima* would be useful to confirm or infirm this theory.

The maximal linear growth rates that we measured for *Saccharina latissima* (2.6 cm · d^−1^) were similar to those measured in earlier studies in this region (3.5 cm · d^−1^ at a site 30 km from ours: Anderson et al., 1981 and 2.3 cm · d^−1^ in the Gulf of St. Lawrence: Gendron, 1989) but were 1.5–4 times higher than in other regions of its distribution (Table 5). Biomass production rates were similar to the values measured in Europe and at the low end of those measured in Nova Scotia (eastern Canada), where exceedingly high rates of biomass production have been reported (14,000 mg · d^−1^; Table 5). This disparity could be explained by short and young kelp individuals tagged at the beginning of our study (~40 cm in length), given the lack of large *S. latissima* sporophytes at this period at the study site (Figure 3; S. Roy pers. obs.). It seems that compared to old blades, young blades tend to first increase in length and width rather than in thickness, possibly, therefore, being outperformed by old sporophytes accumulating biomass faster by elongation, widening, and thickening simultaneously (Koehl et al., 2008). Furthermore, hydrodynamics can have an impact on the blade thickness, with thicker blades usually present in exposed environments (Koehl et al., 2008), which was the case for a few sites used in the comparative studies (e.g., Gilson et al., 2023; Krumhansl & Scheibling, 2011). This biomass production in three dimensions (elongation, widening, and thickening) might also explain the steady growth rates we observed compared to the striking variation in biomass production throughout the summer. Our growth measurements only considered planar growth by blade elongation, omitting the thickening and the widening of the blade, which could explain the disparity between the two measurements.

**TABLE 5:** *Saccharina latissima* growth rates in cm d^−1^and biomass production rates in mg DW · d^−1^.

Region	Climatic region	Growth (cm · d^−1^)	Biomass production (mg DW · d^−1^)	Season	Depth (m)	References
Québec	Subarctic	0.05–2.6 or 1.30 ± 0.08	6–302 or 144 ± 11	June to August	1–5	This study
Québec	Subarctic	0.18–3.5	–	All year	2–6	Anderson et al. (1981)
Québec	Temperate	0.04–2.3	–	All year	4	Gendron (1989)
Nunavik	Subarctic	0.9 ± 0.3	–	All year	8	Sharp et al. (2009)
Alaska	Subarctic	0.05–0.6	–	July–August	5	Spurkland and Iken (2012)
Nova Scotia	Temperate	0.1–0.9	–	May to October	10	Hatcher et al. (1977)
Nova Scotia	Temperate	0.3–1.8	‐	All year	9	Gagné et al. (1982)
Nova Scotia		–	80–14,000	All year	4–6	Krumhansl and Scheibling (2011)
Denmark	Temperate	0.75 ± 0.04	140 ± 120	June–July	7	Nielsen et al. (2014)
Ireland	Temperate	–	150–350	All year	0	Gilson et al. (2023)

### Seasonal primary productivity: Oxygen fluxes and growth rates do not follow the same patterns

Environmental conditions, especially light and temperature, have been suggested in many studies to drive kelp productivity (Pessarrodona, Moore, et al., 2018; Smale et al., 2020; Spurkland & Iken, 2012; Tait & Schiel, 2013). In July and August, our study showed that the strongest gross primary productivity (GPP) rates coincided with the highest mean irradiance and temperature (Table 2), the light explaining a little less than half the variation in GPP (44%) observed during the study. Seasonal variations in light and temperature often intertwine, creating favorable conditions for increased productivity (Anderson et al., 1981; Gilson et al., 2023; Nielsen et al., 2014).

Hatcher et al. (1977) observed that light and temperature explained 63% of the variation in diel net photosynthesis in *Saccharina latissima*. Respiration was affected by salinity, increasing with decreasing salinity. Low and fluctuating salinity can create osmotic stress and affect kelp physiology (Nielsen et al., 2014), especially when salinity is below the optimal range (27–33; Gerard et al., 1987) for *S. latissima*. Kelp in our study site may have been acclimated to local conditions of slightly lower salinities (20 to 24; Table 2). However, drops in salinity to values lower than 16, which were sometimes recorded in our study site, likely affected *S. latissima* physiology (Diehl et al., 2023). From late August to September, an overall decrease in productivity was measured, likely caused by changes in environmental conditions of the light, temperature, and salinity, but also by other environmental drivers given the low percentage of explained variance by those variables. This low explanatory power was especially surprising for temperature, which has been identified in many studies as a the driver of kelp productivity (Andersen et al., 2013; Smale et al., 2020; Tait & Schiel, 2013). This discrepancy is potentially linked to low variation in mean temperatures observed during our study (8–10°C) were in the sub‐optimal to optimal range for *S. latissima* measured in the North Sea (10 to 15°C; Davison, 1987, Davison & Davison, 1987). Moreover, *S. latissima* is known to better acclimate to water temperatures below (Borum et al., 2002; Davison, 1987) rather than above optimal (Wilson et al., 2015), as was the case in our study.

The decline in productivity (e.g., oxygen flux, biomass production) as autumn approaches has been consistently noted in prior studies examining *Saccharina latissima* in the Northwest Atlantic, aligning with findings from related research conducted over similar time frames (Anderson et al., 1981; Chapman & Craigie, 1977; Gagné et al., 1982) or slightly before or after (Gagné et al., 1982; Hatcher et al., 1977; Krumhansl & Scheibling, 2011). Some authors have proposed the existence of an endogenous circannual rhythm in Laminariales dictating the timing of physiological processes (Lüning, 1991; Tom Dieck, 1991) and highly correlated with the photoperiod and irradiance (Chapman & Craigie, 1977; Lüning, 1993; Lüning & Dieck, 1989; Nielsen et al., 2014), especially when nutrients are not limiting (Brady‐Campbell et al., 1984) or when carbohydrate reserves are full (Chapman & Craigie, 1977). *Saccharina latissima* can exhibit different growth patterns depending on the environmental conditions, which have often been attributed to nitrogen availability when light is adequate (Chapman & Craigie, 1977; Espinoza & Chapman, 1983; Gagné et al., 1982). It has been described that under nitrogen sufficiency conditions, *S. latissima* growth follows light availability whereas under summer nitrogen depletion, in winter, growth is limited to periods after mixing events when nitrogen concentration increases, for example (Chapman & Craigie, 1977; Gagné et al., 1982). Our site typically has nitrogen concentrations higher than the sufficiency threshold (10 μmol L^−1^; Chapman et al., 1978; Zhu et al., 2021) during the sampling period (>14 μmol · L^−1^; pers. comm. P. Rioux). The C:N ratio showed in June a value of 14.1 ± 0.1 mol:mol, which is above the nitrogen sufficiency threshold (<10 mol:mol) but still below the nitrogen limitation value (<20 mol:mol; Paine et al., 2023 and references therein). The early August increase in the C:N ratio (20.1 ± 0.1 mol:mol) indicated the onset of nitrogen limitation, but the constant growth rate suggests that *S. latissima* is well adapted to this environment, yielding high growth rates even when nitrogen level seemed to be declining. Nutrient sufficiency, especially in early summer, and adequate light could partly explain the coupling pattern of primary productivity and biomass production simultaneously, as opposed to a decoupling of those processes when nitrogen is limiting (e.g., Nova Scotia; Chapman & Craigie, 1977).

The variation of blade length through time can be seen as a proxy for the kelp state (elongating vs. eroding) and gives insight into whether individuals are producing or losing biomass. The blade length showed a gradual increase until July suggesting that growth was more important than erosion, followed by stable blade lengths in August and September.

In early August, we observed positive growth rates but constant blade length indicating that growth was being offset by blade erosion. As shown in earlier studies (Gilson et al., 2023; Krumhansl & Scheibling, 2012), the erosion of apical tissues, reaching its peak in August in our site (up to 2.0 cm per day; S. Roy, unpublished data), likely contributed to this observed phenomenon. August and September were also characterized by larger variations in blade lengths among individuals, further indicating eroding blades. On some individuals, large pieces were lost, resulting in an overall larger range of blade lengths. The erosion was likely facilitated by a large increase in the abundance of herbivorous gastropods (e.g., *Lacuna vincta*; Montagu, 1803) on the blade from August, a pattern also observed in Nova Scotia (Johnson & Mann, 1988; Krumhansl & Scheibling, 2011, 2012; O'Brien et al., 2015) and Western Australia (De Bettignies et al., 2012). This intensive grazing activity increased blade tissue damage mainly on the blade edges. This coincided with the decrease in growth rates in late August, which could have affected the polyphenol content of the blade. Kelps usually have the highest polyphenol concentration in their tissue and exude high concentrations of phenols during periods of high growth (Schiener et al., 2015), both of which can act as deterrents to grazers (Toth & Pavia, 2002). However, as the polyphenol concentrations decrease with slower growth, the gastropods can overcome this defense and the resulting grazing damage weakens the blade tissue, facilitating the detritus production (Krumhansl & Scheibling, 2011, 2012) and contributing to kelp carbon transfer to higher trophic levels and kelp carbon dispersal (Filbee‐Dexter et al., 2020).

### Carbon budget perspective from daily primary productivity individual measurements

In our study, we noticed that despite having good fixation capacities (NPP), *Saccharina latissima* only assimilated (i.e., BP), BP on average, 18% of the carbon fixed by photosynthesis. Studies on *Saccharina latissima* in the temperate regions of Scotland and Nova Scotia have estimated a higher percentages with from 45% to 50% of the primary productivity going into the storage and growth of new tissues (Hatcher et al., 1977; Johnson & Mann, 1988). Furthermore, the carbon rates estimation for biomass production (11 to 50 mg C · d^−1^) and respiration (−53 to −25 mg C · d^−1^) combined, represented around 30% of the GPP biomass production (181.2 to 353.2 mg C · d^−1^), meaning that a large proportion, potentially up to 70% of the carbon captured was probably released as DOC. It has been observed that exudation of DOC in *S. latissima* can vary from 13% to 35% of the carbon captured (Johnson & Mann, 1988, Johnson & Mann, 1988, Broch & Slagstad, 2012). Dissolved organic carbon released by kelp is often understudied and, therefore, underestimated in the coastal oceans (Paine et al., 2021). A more adequate assessment of carbon released by kelp (respiration, detritus, and DOC) will allow a more complete portrait of carbon flows within, around, and from kelp forest ecosystems and will help in assessessing the kelp sequestration potential better (Pessarrodona et al., 2023). However, the sequestration potential of kelp requires the integration of measurements that are difficult to acquire in situ, such as the lateral export of POC and DOC to other systems, the rapid turnover of kelp biomass, and the complexity of the atmosphere–ocean CO_2_ interactions (Hurd et al., 2022, 2024).

Our comprehensive findings shed new light on the complex interplay of *Saccharina latissima* summer productivity and captured, during this short‐term study, variation in kelp productivity and morphometry, hinting at the effect of seasonal change in environmental conditions on the subarctic kelp phenology. This highlights the importance of seasonal monitoring (Bordeyne et al., 2020) in highly dynamic environments like subarctic regions, which is fundamental for comprehending the vital role of kelp in coastal ecosystems and carbon budgets and as a potential natural solution for contributing to mitigation of climate change. *Saccharina latissima* in the St. Lawrence estuary showed high productivity compared to temperate regions; however, a small proportion is actually used to produce tissues and most of the production is returned to the surroundings. This dynamic could change as the water temperate increase (Wilson et al., 2015) but opens a door on the need to better understand kelp‐associated carbon export, remineralization, and sequestration in subarctic ecosystems.

## AUTHOR CONTRIBUTIONS

**Stéphanie Roy:** Conceptualization (lead); data curation (lead); formal analysis (lead); methodology (lead); writing – original draft (lead). **Christian Nozais:** Conceptualization (supporting); data curation (supporting); methodology (supporting); supervision (equal); writing – review and editing (supporting). **Ladd E. Johnson:** Methodology (supporting); writing – review and editing (supporting). **Fanny Noisette:** Conceptualization (supporting); data curation (supporting); formal analysis (supporting); methodology (supporting); supervision (equal); writing – review and editing (supporting).

## FUNDING INFORMATION

This study was supported by a Natural Sciences and Engineering Research Council of Canada (NSERC) Discovery grant (#RGPIN‐2020‐07065 to FN).

## Supporting information


**Appendix S1.** Photosynthetic surface calculations and allometric relationships.
