# Supplementary material for: Subarctic sugar kelp (Saccharina latissima, Phaeophyceae) summer productivity and contribution to carbon budgets
Source: J Phycol. 2024 Nov 25;60(6):1585–600. doi: 10.1111/jpy.13525 (PMC11670287; doi:10.1111/jpy.13525)
Supplement: Supplementary file 1 — Appendix S1. Photosynthetic surface calculations and allometric relationships. [file JPY-60-1585-s001.zip › jpy13525-sup-0001-AppendixS1.docx]

## Appendix S1 Supplementary materials

1. Photosynthetic surface calculations

Kelp individuals incubated were collected after the incubations. The kelp was laid onto a table with a measuring tape next to it, and a picture was taken. The surface area of the blade was then calculated using Image J. This was done to be able to compare with Hatcher et al. (1977) who used the surface area to express the oxygen and carbon fluxes from photorespirometry incubations.

1. Allometric relationships

Allometric relationships between dry biomass and kelp blade length were determined to estimate individual biomass production throughout the summer. Kelps were collected for this purpose on three occasions: May 27, August 5, and September 12. Twenty to 52 individuals were collected on each occasion by collecting one to five individuals every 5 m along a 50-m-long transect. Individuals were removed by cutting the stipe a few centimeters above the holdfast, and thus biomass estimates included only the blade. Blade length and dry weight (48 h, 60°C) were recorded. Different models were assessed to determine the best regression (linear, power, exponential) to describe the relationship of dry biomass as a function of blade length for each sampling event. Given that the linear and power models were equivalent in May (Akaike information criterion or AIC linear = 49.12, AIC power = 50.69) and August (AIC linear = 146.95, AIC power = 148.65) and that the best model for September was a power model, the latter was selected for comparisons between months, as well as with literature values (Stagnol et al., 2016, Campbell & Starko, 2021; Figure 7).
